# Influence of health rights discourses and community organizing on equitable access to health: the case of HIV, tuberculosis and cancer in Peru

**DOI:** 10.1186/1744-8603-9-23

**Published:** 2013-05-17

**Authors:** Clara Sandoval, Carlos F Cáceres

**Affiliations:** 1 Cayetano Heredia University School of Public Health and Institute of Studies in Health, Sexuality and Human Development, Av Armendariz 445, Lima 18, Peru

**Keywords:** HIV, Public policy, Health rights, Community participation, Access to health care

## Abstract

**Background:**

The right to health is recognized as a fundamental human right. Social participation is implied in the fulfillment of health rights since Alma Ata posited its relevance for successful health programs, although a wide range of interpretations has been observed for this term. While Peruvian law recognizes community and social participation in health, it was the GFATM requirement of mixed public-civil society participation in Country Coordination Mechanisms (CCM) for proposal submission what effectively led to formal community involvement in the national response to HIV and, to a lesser extent, tuberculosis. This has not been the case, however, for other chronic diseases in Peru. This study aims to describe and compare the role of health rights discourse and community involvement in the national response to HIV, tuberculosis and cancer.

**Methods:**

Key health policy documents were identified and analyzed. In-depth interviews were conducted with stakeholders, representatives of civil society organizations (CSO), and leaders of organizations of people affected by HIV, cancer and tuberculosis.

**Results and discussion:**

A health rights discourse, well established in the HIV field, is expanding to general health discussions and to the tuberculosis (TB) field in particular. Both HIV and TB programs have National Multisectoral Strategic Plans and recognize participation of affected communities’ organizations. Similar mechanisms are non-existent for cancer or other disease-focused programs, although other affected patients are starting some organization efforts. Interviewees agreed that reaching the achievements of HIV mobilization is difficult for other diseases, since the HIV response was modeled based on a global movement with strong networks and advocacy mechanisms, eventually succeeding in the establishment of financial sources like the GFATM. Nevertheless, organizations linked to cancer and other diseases are building a National Patient Network to defend health rights.

**Conclusions:**

There are new efforts to promote and protect health rights in Peru, probably inspired by the achievements of organizations of people living with HIV (PLHA). The public health sector must consolidate the participation of affected communities’ organizations in decision-making processes and implementation of health programs. PLHA organizations have become a key political and social actor in Peruvian public health policy.

## Background

The right to health is constitutive of the concept of human rights [1], and hence it has been incorporated into many legislative frameworks. Since the Alma Ata Declaration posited the necessity of social participation to achieve access to health for all [2], it has deemed attention in health policy frameworks and become a common place in official health policy discourse, although the concept is sufficiently broad to warrant diverse interpretations and uncertain practical implications.

### The right to health

The right to health is recognized by the Peruvian constitution [3], but it is treated as a national aspiration rather than as a real entitlement the State should be accountable for, so there is no universal access to care and citizens do not expect free care [4]. While over the past two decades, higher-level health policy statements have addressed health as a right, such approach has not been appropriately operationalized at most lower-level (i.e. specific) health policy formulations, making progress in a practical health rights agenda very difficult. The emergence of a global HIV discourse with a significant health and human rights component, however, has played a key role in influencing a formal Peruvian HIV policy with a health and human rights framework, most clearly expressed in the HIV law reform to warrant universal access to antiretroviral treatment in 2004 [5]. It is uncertain; however, to what extent this health rights-informed discourse has influenced both higher-level and other problem-specific policy formulations.

Peruvian law also recognizes community and social participation in health [6], although such participation has been expressed in mechanisms with limited practical implications, such as the National Health Council [7], or in geographically circumscribed initiatives such as the Local Committees for Health Administration (known as CLAS in Spanish) [8] or NGO-led projects in priority areas (e.g. the Reprosalud project, implemented by a women’s organization with USAID funds [9]). The emergence of the Global Fund to fight AIDS, Tuberculosis and Malaria (GFATM) in 2001, however, which opened the opportunity for national grant applications focused on these diseases, but mandated the constitution of a Country Coordination Mechanism (CCM) with mixed public-civil society participation [10], may have led to some of the most important examples of formal community involvement in ongoing, national-level health decision making processes in Peru, particularly in the HIV field.

### Community participation

In principle health rights are, as all human rights, deemed to be for universal recognition (i). Since Alma Ata, community participation is viewed as a key element in theoretical frameworks formulated for the improvement of people’s health conditions, which will not be achieved solely based on health technology or biomedical advances [2]. As a perspective to enhance health programs through community action, the concept has been applied in diverse ways, sometimes referring to the empowerment of populations, and other times encompassing strategies to link populations to health services, e.g. through promoting visits to health services among community members, or choosing community representatives to a governmental health committee, or partnering with NGOs or to implement a strategy of community health promoters [2]. Participation refers to a distribution of power relations in health processes and it is important to examine how this process takes into account in different health issues or diseases [11].

People and communities have the right to participate in a free, meaningful and effective way in decisions about the health policies or programs that affect them, and such participation is one of the most important principles to achieve the right to health [12]. Relations between policy makers, health providers and citizens are important to achieve improvements in the health systems leading to an inclusive and sustainable social and health policy [13]. Social participation in health depends on: a) the context in which it is enacted; in other words, the institutional and political context in which actors interact and their participation takes place: NGOs, affected communities, the public health sector and the specific role of civil society, b) internal factors including the characteristics of the civil society organizations (NGOs, affected communities and others), and the performance of their leaders; and c) the role of state public policy [14]. Since the 1990s, NGOs started to work together with the government in specific disease-focused programs and/or in some regions in Peru; however, rather than taking part of policy decision making, they mostly saw their role limited to implementation of some projects [15].

### HIV/AIDS, mobilization and universal access

HIV/AIDS mobilization demands universal access to prevention, care and treatment, and confronts stigma and discrimination of affected people [15]. Due to the characteristics of HIV transmission and the presence of global HIV initiatives, the disease has shown an unprecedented dynamic involving governments and civil society globally. In today’s world, the emergence of HIV/AIDS is an example of a cultural product which reflects global changes [15-17].

Towards the beginning of this century, the contrast between accessibility of ARVT in higher income countries, and lack thereof in most of the global South, became morally unacceptable. The UNGASS commitments promoted the creation of a Global Fund to help countries fight AIDS, Tuberculosis and Malaria, diseases that were causing a very large number of deaths in the South [18].

In the case of Peru, receipt of four Global Fund grants to support HIV activities, and other three to support TB activities made the country a particularly relevant focus of analysis of how public discourse as well as community mobilization around these diseases was (or not) affecting the general health rights discourse and practices [19].

We aimed to assess whether globalized health rights discourses and models of community participation linked to the HIV response have had and/or may still have an impact on broader health policy formulations, and on both programmatic and implementation aspects of programs focused on other chronic or catastrophic diseases (e.g. cancer). More specifically, this study explores to what extent health rights discourse and mobilization on HIV/AIDS has influenced the perceptions and discourses about the right to health, equitable access and social participation in other areas of public health in Peru. We analyze this influence in the context of two specific health conditions: tuberculosis and cancer, where the first one is also a focus of grants from the Global Fund in Peru, while the second is not part of the Global Fund mandate. Cancer has some characteristics that allow for comparison with the other conditions: a stigmatized, chronic disease with a costly treatment, and the existence of an identifiable constituency of affected people. An initial attempt to also include other chronic health conditions such as diabetes and chronic renal insufficiency failed because the level of organization around those conditions is either almost non-existent (e.g. diabetes) or circumstantial/occasional (e.g. when a problem emerges in provision of dialysis), so a decision was made to focus the study on HIV, tuberculosis and cancer.

## Methods

### Research questions

We aimed to analyze (1) to what extent globalized discourses about health rights, access with equity and social participation adopted by local actors involved in the HIV/AIDS response have influenced other discourses on health access and health rights in Peru; and (2) to what extent HIV-related processes and networks constitute a model for processes and interactions involving both the State and Civil Society (including rights organizations, vulnerable population advocates, and academic institutions) with regard to other public health problems and to general discussions around access to health. Since stakeholders are likely to have contrasting views about these topics, we used stakeholder analysis [20] to collect and analyze information about discourses and mobilization on health rights among general and disease-specific stakeholders in Peru. This framework allows to explore their intentions, interrelations, agendas and the influence or resources they have brought – or could bring to decision making processes A comparison between the three different processes is also possible.

### Data sources

We identified and gathered key official documents concerning health policy in general and around tuberculosis, HIV/AIDS and cancer, including: (a) National Health Plans; (b) Disease-specific programmatic documents, including the Strategic Multisectoral Plans for HIV and tuberculosis; (c) projects funded by the Global Fund; and (d) other relevant documents. These were identified at the Ministry of Health official website, the website of the GFATM; and through references from interviewees.

We also identified and listed general and disease-specific stakeholders, including government actors, affected communities and Civil Society organizations. Out of an initial list of 20 individuals (10-government, 10-civil society), we interviewed 17 people involved in general health policy and in the national responses to HIV, tuberculosis and cancer, including government officials (8), and leaders of NGOs and affected community organizations (9). This list was balanced with regard to government and various viewpoints among civil society organizations.

We elaborated a semi structured interview guide covering the following topics: National health policy and the formal status of health rights and community participation; characteristics of health programs in HIV, Tuberculosis and Cancer, and level of community participation; agency of NGOs and community organizations; leadership; links of health mobilization with Peruvian history and civil society participation in health.

### Data collection and analysis

After obtaining informed consent, semi structured interviews were conducted by one of the researchers, (Clara Sandoval Figueroa) in a private space that ensured confidentiality. They were recorded with subjects’ permission and subsequently transcribed.

Use of Stakeholder Analysis [21], helped generate knowledge about the actors’ perspectives on each particular topic. The transcripts were read by the two researchers according to the research questions. Paragraphs providing key ideas were segmented and interpreted on the basis of the thematic categories guiding the study. These fragments were jointly interpreted by the researchers, and some were selected to illustrate the findings and conclusions of this text. Policy documents were read by the two researchers and analyzed for content according to the research questions; they were considered as statements of the official government standpoint.

Information in interviews was codified according to preliminary study categories derived from the research questions and considered in the interview guide, i.e. national health policy, the right to health, social participation in health, participation of affected communities in policy and programmatic aspects of the three focus conditions, health program components, leadership, social mobilization and activism, influence of HIV activism in policy outcomes concerning tuberculosis and cancer, among others.

### Ethical integrity

The full proposal was reviewed and ethically approved by the Cayetano Heredia University Independent Review Board, including informed consent forms to be used to allow for data collection from participants. The informed consent forms explained purpose, procedures, risks and benefits of participation, and asked for participant’s consent to be interviewed, to have the interviews recorded and transcribed, and to use specific quotes in the study report, without identifiers.

## Findings and discussion

### Policy context and key documents

The Peruvian government has recognized various international human rights Conventions and Declarations that acknowledge the right to health; economic, social and cultural rights; civil and political rights; and the elimination of all forms of violence against women, and of all forms of racial discrimination [20]^ii^. Recently, major policies have been officially established to bridge the gaps of health access and set up comprehensive health insurance. In January 2010, a Universal Health Insurance Program, still to be regulated and implemented, was launched by the Ministry of Health of Peru [22], promising that, in the years to come, all Peruvians have access to a prioritized package of health benefits and hence minimize out-of-pocket health spending [23,24]. Implementation of a policy for health access to all will, however, take significant time and, arguably, cannot be limited to financing mechanisms; rather, it implies a transformation of health systems and models of care that accounts for barriers that are not related to cost.

In parallel to a general discussion on health access over the past 20 years, which included a sanitary reform following the Washington Consensus [25], an intense yet somewhat isolated discourse has emerged concerning HIV and the right to health over the past decade, advocated for by civil society. The national response to HIV/AIDS in Peru has involved for the first time a number of stakeholders not previously participating in the health field. Notably, the social meanings attached to the epidemic forced the national health system to hear other voices. Those new voices included People Living with HIV/AIDS (PLHA), members of traditionally excluded most-at-risk populations (i.e. sex workers, sexually diverse populations), representatives of professional NGOs working on HIV/AIDS, academics and other actors such as faith-based institutions. Moreover, the implementation of projects funded by the GFATM, which started in 2004, generated a new dynamic in prevention work and, more importantly, led to the rollout of the antiretroviral treatment program, with a commitment from the government to fully assume its costs from 2006 onwards, formalized in a reform of the 1996 HIV law [26].

Part of this HIV-specific discourse has made its way onto a broader health access discourse, articulated by Forosalud, the Civil Society Health Consortium. In the core of this new discourse was, in fact, a plea for participation of those new constituencies whose presence started to be seen as necessary [27]. Importantly, this discourse posited the need to redefine the relationship between providers and “patients” – now to be considered affected constituencies, emphasizing the importance of quality of care, the need to adopt a comprehensive health care perspective (including sexual and reproductive health) and to achieve access for all (including PLHA and vulnerable populations, previously invisible to the system).

This HIV-focused discourse is expressed in official documents such as the National Multisectoral Strategic Plan, the HIV grant proposals submitted to the GFATM, and the HIV/STI Technical Guidelines (e.g. for comprehensive care of PLHA, for prevention activities among vulnerable populations) [28-30]. In theory, these documents contemplate health as a human right, to be attained without distinctions, as well as the recognition of global public goods, which include health in the pursuit of human development.

Changes at the level of discourse had a correlate at the level of community participation. In 2001, the Peruvian government submitted an HIV proposal to the GFATM Funding Round 1, which failed on grounds of limited evidence of community participation. The government’s decision to re-apply in Funding Round 2 (2003) implied acceptance of GFATM’s conditions, including the formation, as the national counterpart, of a Country Coordination Mechanism (CCM – called CONAMUSA in Peru) [31], with participation of various government sectors, organizations of affected and vulnerable populations, AIDS service organizations and research institutions. This proposal was successful, as were three other HIV proposals in Funding Rounds 5, 6 and 10 (as were TB proposals in rounds 2, 5 and 8) [29,31]. This confluence led to redefinition of the relationship among sectors. While multisectorality in CONAMUSA is still incipient, it is a new experience in the health sector. Stakeholders value multisectoral work but recognize that much still needs to be defined [21]. Vulnerable populations and PLHA consider that their transition from ‘patients’ to members of a deliberating body has changed their role and the way they are treated by other actors who used to have a monopoly in decision making. Discussion and negotiation amongst stakeholders have been incorporated as part of the regular practice in decision making processes. While many actors consider HIV/AIDS as mainly a health issue, they have recognized that the national response must consider other dimensions of the epidemic; consequently, it is now acknowledged that civil society organizations including those of affected people/families and vulnerable populations have to play a key role in this response. Initial steps have been taken in this direction, although many activists still consider that truly horizontal collaboration is an ideal model, far from the present arrangement where many of the real-life inequalities persist [10,19].

We identified policy documents that regulate the national responses to the three conditions of the study, and found that the responses to HIV and TB are, in theory, regulated by the National Multisectoral Strategic Plan for HIV/AIDS (HIV/AIDS PEM) and the National Multisectoral Strategic Plan for Tuberculosis (TB PEM) respectively [28,32]. These documents recognize the participation of civil society organizations including those of affected communities. Moreover, these strategic plans have a decentralist stance, focusing on political administrative regions. While the extent to which those plans have been validated at the regional level is not clear, such plans offer the regions some guidelines to begin working according to their specific social context [28,32].

Concerning the national response to cancer, we must refer to the National Plan for the Strengthening of Cancer Prevention and Control, formulated in 2006 by a coalition including the Ministry of Health (MoH), the National Cancer Institute (INEN) and a number of academic and professional institutions [33]. In that document no mention is made of the right to health, but it is recognized that cancer is a key cause of mortality, in part due to limited access to treatment. From a public health point of view, this plan proposes a number of strategies for the next ten years (2006–2016); participation of affected communities is mentioned only in terms of promoting the association of cancer survivors. Unfortunately, after its formulation, such plan was left aside after some political changes at the MoH^iii^. Then, INEN focused mostly on clinical work and, together with other MoH divisions, such as health promotion, on a few preventive activities for certain cancers, such as anti-smoking campaigns, and promotion of periodic screening for cervical cancer [34], breast cancer and prostate cancer, according to age.

The 2007–2011 HIV/AIDS PEM went through a political process that gave at least partial legitimacy to a social response process – there was an attempt to include some regional actors at certain points. This plan makes frequent reference to the 2001 UNGASS Commitments [19] and the Millennium Development Goals [35]. Interestingly, it reads as a multisectoral public policy with a weak link with other national public health policies, in contrast with a strong link to the global HIV political and technical framework (MDGs and UNGASS). This portrays the strong influence that the globalized HIV/AIDS discourse has played upon the national HIV response in Peru.

The TB Multisectorial Strategic Plan [32] recognizes that a national response implies collaboration between civil society and the public sector [36]. The plan incorporates social actors’ participation of NGOs and affected organizations. The history of the tuberculosis response in Peru is not characterized by the role of a vibrant movement as observed in the HIV/AIDS response. Before the onset of support from the Global Fund only a few social actors showed a relevant trajectory of commitment to increase access to treatment, mainly linked to the Catholic Church, in addition to international NGOs such as *Partners in Health*. After the implementation of the TB Global Fund projects, a substantial change was observed, with new affected communities’ organizations participating in the response.

Concerning cancer programs, organized groups of affected individuals such as the *Club de la Mama* (Breast Club), formed at the National Cancer Institute (INEN) have been working to deliver information about prevention and treatment so as to improve the quality of life of people with breast cancer [37-39]. Other associations are linked to volunteers who work helping people in extreme poverty who suffer cancer, especially children and people who do not live in the Capital where the National Cancer Institute is located. In the last few years a NGO concerned about unequal access to cancer treatment, Esperantra, had an active participation to push for universal access to treatment through the Comprehensive Insurance System (SIS) [40].

Table 1 summarizes the context of the current situation of the three health conditions addressed in the study. We see differences related to the presence of national and international funding sources, the establishment of international activist networks, and the existence of National Multisectoral Strategic Plans (PEM, for its initials in Spanish) that take into account the participation of affected communities.

**Table 1:** Key aspects of context and responses to the three diseases

**Health condition**	**Presence of international financial support**	**Funding from the Peruvian National Treasury**	**Networks and mobilization**	**National policy documents and official status of community participation**
**HIV/AIDS**	Global Fund Projects. Until 2006 funding included prevention and treatment, and later it focused only on prevention and multisectoral work.	National Treasury (public funding) covers cost of treatment, as mandated by law.	A strong global movement. Peruvian movements existed before the emergence of GFATM, and became involved in governance & implementation	National Multisectoral Strategic Plan (PEM); National HIV/STI Strategy Guidelines. Community participation is defined in official documents.
**Tuberculosis**	Global Fund Projects mainly focused on prevention and multisectoral work and multi-drug-resistant TB treatment.	National Treasury (public funding) covers cost of treatment, as mandated by law; difficulties with multi-drug-resistant TB.	No significant mobilization around the world. A few associations borne in Peru before Global Fund.	National Multisectoral Strategic Plan (PEM);National TB Strategy Guidelines. Community participation is defined in official documents.
**Cancer**	Some private financial support to ensure access to treatment. Then, only international clinical trials of the pharmaceutical industry.	Part of the treatment cost covered by the national health insurance when patients are poor and lack social or private insurance. Otherwise treatment cost covered by patients or supported by charity.	Movements around the world for prevention and/or early screening. No unified movement exists here; only early patient groups (INEN) & 1 NGO (Esperantra)	No specific documents about social participation in cancer. However a Strategic Plan to enhance Cancer Prevention and Control was formulated for 2006–2016 with limited implementation.

### Mobilization in HIV/AIDS, tuberculosis and cancer

Generally, actors believe that the international political and social process that accompanied HIV/AIDS since the beginning of the epidemic created a very special context for the social response to it. They mentioned that activist movements of PLHA emerged from previous ones focused on gay rights, since most people affected in Peru since early in the epidemic were men who had sex with men. PLHA activists stated that, while this association between HIV and the gay community led to increased stigmatization of this group, it allowed people affected by the disease to organize, not only as a group in need, bus as bearers of a social condition attached to a strong identity. According to a government official, another important factor for the success of PLHA organizations in becoming a global movement was the fact that HIV was seen as an emerging pandemic without national geographic frontiers. Hence, probably both sources: the local history of gay activism, and the worldwide mobilization to confront a pandemic, were important in the emergence of a health rights discourse.

It has been based on people living with HIV and in particular it has relied on gay activism, and interested groups that have resources that other diseases do not have, not even tuberculosis or cancer. There has been a strong worldwide pressure with groups of power that have lobbied for the importance given to this disease, (although) every disease is important and people affected by any disease are important too. It has been an extremely strong pressure for countries to take action and when they have not been able to take action due to lack of resources, new resources have been created such as the Global Fund*.* (Representative, Comprehensive Health Insurance System in Peru).

Therefore, since its early stages, HIV/AIDS was presented as a global disease posing an urgent need to generate financial resources, particularly to avoid gross inequities between the global North and the global South. PLHA activism was reinforced by global networks. The global HIV/AIDS movement was able to generate the creation of the GFATM, as a multilateral financial source to fight not only the HIV pandemic, but also two diseases responsible for many deaths in the global South: TB and malaria. One of the most important advantages of the Global Fund was its capacity to fund projects with a great economic impact. Various authors posit that a worldwide globalization process implies breaking barriers such as frontiers, communication and others [15-17].

Concerning tuberculosis, all actors stated that national organizing around this disease had started way before the Global Fund projects, but with results not nearly as successful as those observed in HIV/AIDS mobilization.

Look carefully: before the Global Fund, the national TB response involved important sources of cooperation from national NGOs and the Peruvian government. Historically that program has been in place for a longer time and was well organized at the primary care level. However, the HIV/AIDS response showed a more powerful dynamic with international resources. (Civil Society Representative, Forosalud).

No such community mobilization has existed for cancer before, probably given the catastrophic rather than chronic nature of many cancers, as well as the diversity of both neoplasms *per se* and people affected by them. In fact, well structured health activism faces considerable constraints concerning cancer, for various reasons: diverse survival rates (for example, breast cancer vs. pancreas cancer); the acute, sometimes incapacitating evolution of cancer and its treatments; and diverse social origin of people affected by various cancers (e.g., people with middle or high income treats at private clinics and people with low income at national hospitals or discontinue treatment, these diverse situations in people affected by cancer hinders their activism), determine distinct concerns among people affected. Conversely, greater homogeneity is apparent among populations affected by the other conditions who get organized (i.e. HIV activists tend to be young or middle-aged, lower-middle income women and non-heterosexual men; TB activists are usually affected by poverty) [41]. Quantification of such differences is beyond the scope of this study; nevertheless, the limited amount of information available warrants the need for epidemiologic surveillance, access and needs should become a priority for the health sector.

As previously pointed out, only the most common conditions were a focus of preventive activities led by the MoH, but treatment support was largely based on charities, and two other factors emerged: private cancer insurance (individually focused and very popular) and recruitment of lower-income patients in clinical trials of the pharmaceutical industry, where concerns about the conditions in which informed consent is requested have been raised [37]. Emerging cancer activists look the PLHA experience with interest because PLHA gained access to medication and treatment as a health right; they wonder why this is not happening for people affected by cancer:

We always think about access to cancer treatment, which is a problem similar to HIV in the sense of treatment costs…, but we ask why there is no free access to treatment in cancer and why is there no Global Fund for the cancer response? I consider equity in access to health as a human right. (Representative of emerging Cancer NGO).

While international funding is a key factor in social participation of people living with HIV, such participation pre-dated the emergence of the Global Fund. Moreover, activists and people affected by tuberculosis and cancer consider that HIV mobilization had a clear influence on other initiatives to demand the fulfillment of health rights, particularly concerning access to treatment. This seems to have encouraged the health sector to prepare a Plan for Access to Treatment for the Poor [42]. Moreover, it seems that social participation in policy processes for these diseases cross-fertilizes and supports a general path towards greater state accountability in matters of access to health, in spite of the contrasting levels of achievement across movements. We can analyze in their Multisectorial Plans different levels of social participation, such as a considerable high level participation of HIV organizations, middle participation in Tuberculosis which have less organizations across the country and cancer organizations focus in deliver prevention information [28,32,33].

### Participation of affected communities

Representatives of the national TB response consider that the presence of tuberculosis representatives in the Peruvian CCM (CONAMUSA) has been a very good experience. Tuberculosis leaders consider that they can take advantage of the know-how of HIV leaders; and recognize the role played by the HIV movement even before the presence of the GFATM:

So that has made people affected by tuberculosis start to look at this empowerment of HIV organizations and try to replicate it, because this empowerment will allow decision-makers to become more concerned about an illness. I think that previous HIV work, from the 1980s, has strengthened us, allowing TB representatives to start incorporating lessons from the HIV experience. (Government Official, MoH).

The influence of discourses about participation of affected communities through their organizations is appreciated by representatives of the Ministry of Health, valuing lessons learned from the HIV/AIDS field where they feel that a “strategic partnership” was established between government and civil society. It is further recognized that the dynamics of the HIV participatory approach has provided new strategies to this new partnership:

We see in the case of people affected by tuberculosis, that we can learn from the HIV experience at the spaces where we have been able to work together, they have more opportunities, they have a good level of training as representatives, much better than people with tuberculosis. (TB Movement Representative).

For one of the new emerging cancer activists, who officially does not work with leaders of the HIV response, such response is considered almost unattainable.

As mentioned above, WHO has highlighted the importance the participation of affected communities in the decision-making process on health and in the sustainability of health systems. The participation of organizations affected by tuberculosis has been encouraged by the influence of HIV activism and the empowerment HIV activists experienced through their legitimization as stakeholders in the HIV response:

Global Fund projects incorporated organizations and mobilization from civil society working on HIV and tuberculosis. Before the Global Fund there was treatment, but with the Global Fund Projects, organizations of people with tuberculosis pressed the Ministry of Health to improve the response with the financial support that was being received at that time. Then that empowerment moved us forward as well (Representative, National Health Insurance System).

The national response to cancer has been much poorer. One substantial difference is that there is no substantial funding for cancer care, and that a large part of costs has to be covered out of pocket if the patient does not have private insurance coverage.

With the experience of HIV, we thought that it is important for the next year that a person with cancer or in remission becomes the President of our group. Because I am the president but I am a doctor at the Hospital. The idea is to get, like for HIV and tuberculosis, more financial support, and make it possible that patients participate more. (Medical promoter, “Club de la mama”- Breast Cancer Club).

As stated above, fulfillment of the right to health is considered to imply, in most cases, the participation of affected communities in decision making process [2]. Interviewees from the community are now all in agreement that the State has the obligation to provide treatment to all people. Mobilization related to access to treatment as an expression of the right to health is the most important lesson that cancer representatives have learned from HIV activism; while for tuberculosis patients, the role of skilled participation of affected communities is the most important message they got from the HIV movement.

It is also relevant to ask whether any experience in the HIV movement should not be replicated by other health movements. While activists in other movements are not aware of details, HIV activists were critical about the problems emerged in their movement when funding became available through the Global Fund grants. As funding was provided through consortia that should include participation of PLH organizations, the Global Fund processes generated fragmentation and disruption of the meager social capital they had been able to accumulate in the early 2000s, with consequences over their demands for access to universal treatment and equal access to most vulnerable populations [43].

Still, respondents consider that investments in HIV observed globally can hardly be matched for other diseases, particularly in research:

I doubt that, in research, in the last fifty years there has been innovation in TB treatment as it has been in HIV therapies… not even in the last five years. I do not know of any. What about progress in research on cancer and the different types of cancer that may affect people? There has been some activity leading to improvements among some private organizations, philanthropic entities or research institutions at universities, but all of this with partial funding. There is no unification of funds or efforts around issues other than HIV/AIDS. I think that tuberculosis programs are funded by the Global Fund because of the co morbidity with HIV/AIDS. I do not know other cases like this one (Official, MoH).

Cancer activists consider it unfair that PLHA have access to free treatment and cancer patients do not. For TB and cancer activists, some of the difficulties they identify in equaling the achievements of HIV/AIDS mobilization include: lack of trained activists (in both groups); less political experience (particularly among TB activists, who share some spaces with HIV leaders), and lack of a perspective of access to health as a right (particularly among cancer activists).

It is difficult because affected people do not feel prepared to defend their rights. (Activist, TB movement).

Then you realize that more than 70% of those affected by cancer don’t recognize health as a right, that people have the right to demand many things related to health, I mean, to demand quality of care (Activist, cancer movement).

However, costs of cancer treatment are usually high, particularly the newest and most effective. This is not the case for HIV and tuberculosis, where standard regimes based on generic drugs are available and effective. Recently, a national debate was held in the press when the pharmaceutical distributors abstained from responding to a government call for bids to purchase a large number of cancer treatments at lower prices, to avoid a fall in their high revenues; this motivated the direct import of medicines from international distributors [44-47].

In addition, TB activists stated that people with tuberculosis do not want to be identified; they are not prepared to assume a role as leaders and feel unable to talk with authorities.

People Living with HIV have the courage to come out. People with tuberculosis don’t do that. (Activist, TB movement).

Another difference they perceive is related to the characteristics of treatment: tuberculosis treatment usually lasts six months, and people want to reintegrate into their activities as soon as they can. Therefore activists and people affected by this disease move away from their organizations and networks when they finish treatment in order to continue personal activities. While people with cancer may experience rapid health deterioration, PLHA have medical check-ups and early treatment so activists may engage in activism for a longer time with relatively good health.

Only a few people can commit long term; they return to their normal life, we cannot force them. (Activist, TB movement).

Patients with cancer may experience rapid health deterioration because of the disease or treatment; these factors don’t let them engage in activism. (Health Ministry Official).

Therefore, specificities of the experience with tuberculosis and cancer create an environment where replicating the HIV/AIDS experience in activism and mobilization is rather difficult. They lack the international networks and support to receive training in important issues such as health rights and to share experiences globally; and treatment consequences are an obstacle to do activism for people affected by cancer.

However, both cancer activists and Forosalud show enthusiasm in relation to the recently created National Patient Network. People affected by several chronic diseases including PLHA organizations participate in the Network in order to defend patients' health rights. They seek to articulate the organizations affected by specific pathologies, looking for patients to become agents of change to improve not only access to treatment and quality of care in health services, but also to empower patients to promote changes in the health system and in medical training, monitoring the delivery of medicines and improving access and quality of care for those affected.

The patients are organizing themselves, all the groups of chronic diseases, people living with HIV, people with cancer, chronic renal failure, diabetes, etc. The idea is to give a level of articulation because they have common demands. We all have suffered the problems of lack of security and quality of services, appropriate treatment services, long waiting times, lack of information or informed consent (Representative, National Patient Network).

## Conclusion

In this study we have analyzed to what extent discourses on equitable access and health rights developed by actors involved in the HIV/AIDS response have influenced other discourses on health access and health rights in Peru; with a focus on tuberculosis and cancer.

Overall, there is recognition of the importance of social participation and mobilization on health rights among government and community actors; nevertheless, representatives of tuberculosis and cancer organizations claim that it is imperative to reach those achievements in the particular cases of those diseases. At the same time, those and other chronic conditions face particular obstacles that are either intrinsic to their natural history or severity, or to the lack of a perception that access to care for them should be a right for which the State is to be held accountable. A more critical difference is the fact that social and political HIV/AIDS processes have been guided by a global movement with both bureaucratic and community components, that supports a worldwide mobilization to defend PLHA health rights in various ways, including financially.

While discourses on health access, health rights and participation of community based organizations have expanded to the general discourse on health and to other disease-specific policies, cancer community stakeholders have not developed mechanisms for horizontal dialogue with the State, and their organizations remain fairly medicalized and linked to the medical facilities.

In conclusion, HIV-related health rights discourse and community mobilization have played an influence in health policy in general and in at least two specific chronic diseases where access to care has historically been limited. It is still critical to stimulate discussions on the role of the State in access to health for all (i.e. considering health rights not as an aspiration but as an entitlement the State must become accountable for; and taking the steps that can make it possible). It is also important to facilitate the participation of affected communities in decision-making processes and in the implementation of health programs to improve quality of care and access to adequate treatment. Concerning civil society mobilization, the recent initiative to create a national network of patients to defend health rights is a promising effort that some NGOs and community based organizations are trying to move forward, inspired upon the achievements of Peruvian PLHA organizations. Ironically, HIV mobilization achievements have created a gap in equitable access to care in Peru, one which the health sector will need to solve in the near future.

### Endnotes

^i^ According to the World Health Organization (WHO), “every country in the world is now party to at least one human rights treaty that addresses health-related rights. Concerning the right to health, WHO declares that *"the enjoyment of the highest attainable standard of health is one of the fundamental rights of every human being without distinction of race, religion, political beliefs or economic or social condition”.* The right to health is recognized in various international and regional human rights treaties and conventions around the world [2,12], including the International Convention on Economic, Social and Cultural Rights (1966), the Convention on the Elimination of All Forms of Discrimination against Women (1979) and the Convention on the Rights of the Child (1989).

^ii^ The current Constitution of Peru, sanctioned in 1993, recognizes the right to the protection of health in its Article 7; and in its Article 9 it proclaims the State as responsible for setting a national health policy [2]. The General Health Law ensures the application of equal access to health services [23].

^iii^ Only in July 2012 a New Cancer Treatment Program has been launched by the President, which implies amplification of the comprehensive health insurance portfolio. Details of its implementation are still to be announced.

## Abbreviations

AIDS: Acquired Immune Deficiency Syndrome; CCM: Country Coordination Mechanism; CONAMUSA: Peruvian CCM; CSO: Civil Society Organization; GFATM: Global Fund to fight AIDS, Tuberculosis and Malaria; ARVT: Antiretroviral Treatment; HIV: Human Immunodeficiency Virus; MDGs: Millennium Development Goals; MoH: Ministry of Health; NGO: Non Governmental Organization; PLHA: People Living with HIV/AIDS; TB: Tuberculosis; UNGASS: United Nations General Assembly Special Session; WHO: World Health Organization.

## Competing interests

We declare that we have no competing interests.

## Authors’ contributions

CS conducted this study as a component of a larger study, drafted and wrote the manuscript; CFC was the Principal Investigator of the larger study in Peru, reviewed and oversaw the manuscript. All authors read and approved the final manuscript.
